# Horizontal federated learning and assessment of Cox models

**DOI:** 10.3389/fdgth.2025.1603630

**Published:** 2025-06-12

**Authors:** Frank Westers, Sam Leder, Lucia Tealdi

**Affiliations:** ^1^ Applied Cryptography & Quantum Applications, Netherlands Institute for Applied Scientific Research (TNO), The Hague, Netherlands; ^2^ Data Science, Netherlands Institute for Applied Scientific Research (TNO), The Hague, Netherlands

**Keywords:** Cox regression, federated learning, multiparty computation (MPC), privacy enhanced technologies (pet), survival analysis, open-source software

## Abstract

The Cox Proportional Hazards model is a widely used method for survival analysis in medical research. However, training an accurate model requires access to a sufficiently large dataset, which is often challenging due to data fragmentation. A potential solution is to combine data from multiple medical institutions, but privacy constraints typically prevent direct data sharing. Federated learning offers a privacy-preserving alternative by allowing multiple parties to collaboratively train a model without exchanging raw data. In this work, we develop algorithms for training Cox models in a federated setting, leveraging survival stacking to facilitate distributed learning. In addition, we introduce a novel secure computation of Schoenfeld residuals, a key diagnostic tool for validating the Cox model. We provide an open-source implementation of our approach and present empirical results that demonstrate the accuracy and benefits of federated Cox regression.

## 1 Introduction

Survival modeling is a common type of data analysis; it aims to predict the time until some event occurs based on historical data. In healthcare, this is often used to study the influence of certain covariates - such as biomarkers or drug use - on the occurrence of some adverse event. The Cox proportional hazard model is a common model for survival analysis in medical research. However, researchers do not always have enough data available to reliably fit a Cox model. Combining the data of multiple medical centers or other parties, such as health insurance providers, would help address this issue, but is often not possible due to privacy restrictions or legislation. This leads to less accurate models and predictions or to potentially useful covariates being ignored.

Federated learning can be used to fit statistical models on distributed data. It allows multiple parties to fit a model without sharing the underlying data. Only certain statistics are shared. However, one of the requirements of federated learning is that the loss function is separable, which is not the case for the Cox model. In this paper, we show a solution for training a Cox model on horizontally partitioned data. Horizontal partitioning here means hospitals have datasets with different patients, but the same type of data on each patient. In addition, we show how to securely compute the Schoenfeld residuals on horizontally partitioned data, which can be used to assess a model computed using federated learning. Finally, we provide a comparison between these methods and existing (centralized) implementations.

The organization of this paper is as follows. In Section 2 we describe the methods used: the background of the Cox model, federated learning, the survival stacking technique, logistic regression in a federated setting, as well as Schoenfeld residuals and our secure approach. Then in Section 3 we present the main results of our research; our objective is to quantify the benefits of federated Cox regression and compare different optimizers. Finally, in Section 4 we discuss our results in the context of prior research, give limitations of our work, and formulate further research questions.

## 2 Methods

### 2.1 The Cox model

The Cox Proportional Hazards (CPH) model, introduced by Cox in 1972 (1), is a widely used statistical method to analyze survival data. It aims to quantify the relationship between survival time and one or more explanatory variables, known as covariates, without requiring strong assumptions about the baseline hazard function. The model is particularly useful in medical and epidemiological research to study the effect of covariates on survival outcomes.

The Cox model expresses the hazard function, which describes the instantaneous risk of failure at time t for an individual with a given set of covariates, as:

$$\lambda (t|Z)=\lambda_{0}(t)\cdot e^{\beta Z}=\lambda_{0}(t)\cdot e^{\beta_{0}Z_{0}+\beta_{1}Z_{1}+\cdots +\beta_{p}Z_{p}}$$

Here $Z=(Z_{1},\ldots ,Z_{p})$ represents the vector of covariates and β are the corresponding regression coefficients. $\lambda_{0}$ is the baseline hazard function, representing the hazard when all covariates are zero. The key feature of the Cox model is its proportional hazards assumption: the hazard function for different individuals is proportional over time. If a coefficient $\beta_{i}$ is positive, the corresponding covariate $Z_{i}$ increases the hazard, which means a higher risk of failure. Conversely, a negative $\beta_{i}$ implies a protective effect, which reduces the hazard.

A major advantage of the Cox model is that it does not require a specification of the baseline hazard $\lambda_{0}(t)$, making it a semi-parametric model. To estimate the coefficients β, the Cox model maximizes the *partial likelihood*. Suppose we have a data set $D=\left(Z_{i},t_{i},\delta_{i}\right)$, where $Z_{i}$ are the covariates, $t_{i}$ is the observed survival time, $\delta_{i}$ is the event indicator (1 if an event occurred, 0 if censored) for individual i. Then, the partial likelihood is given by:

$$\begin{array}{rl} L(\beta ) & =\prod_{i\in D;\delta_{i}=1}P(i\text{ fails}\;|\;R(t_{i}))\\ & =\frac{\exp(Z_{i}\beta )}{\sum_{\;j\in R(t_{i})}\exp(Z_{j}\beta )} \end{array}$$

Here, $R(t)=\{i|t_{i}\geq t\}$, that is, the set of individuals still in the study at t, or *risk set*. Maximizing this likelihood leads to estimates of the β coefficients, which quantify the effect of covariates on survival. The statistical significance of these estimates can be assessed using hypothesis tests such as the Wald test, p-values, or likelihood ratio test. The Schoenfeld residual test can be used to test whether a data set satisfies the proportional hazard assumption.

### 2.2 Federated learning of Cox models

One limiting factor in medical research is often the availability of reliable patient data. In addition, these data are often restricted to a single institution or region, resulting in datasets that may lack diversity and generalizability. Privacy concerns and regulatory constraints make it difficult to share sensitive health data between institutions. These challenges have spurred research into privacy-preserving methods that enable collaborative studies while protecting patient confidentiality (2).

Federated Learning (FL) is one such method that allows multiple institutions to collaboratively train a model without sharing raw data (3). In federated learning, the data remains decentralized, and only model updates, such as gradients, are exchanged between institutions. The federated learning process generally consists of three steps:

1. Each participating institution computes an update to the model using its local data and sends this update to a central aggregator.
2. The aggregator combines the local updates to produce a global model update and distributes it to all participants.
3. Each institution updates its local model with the new global update. This process is repeated until a stopping criterion is met.

A common approach to aggregation is averaging, which is also used in this work. For updates, we use the gradients in a gradient descent optimization process. It is important to note that some information, namely the gradients, is being shared with the server. In cases where this is a problem, the server can be replaced by a secret-sharing scheme of a form of homomorphic encryption or differential privacy can be used.

Although federated learning appears to be a natural fit for Cox regression — where each institution computes gradients with respect to the partial likelihood and the gradients are averaged for a global update — this approach is not straightforward. The reason is that the partial likelihood in the Cox model involves summing over all individuals in the risk set at a given time. Since each institution only has access to its own local dataset, this value in the partial likelihood cannot be accurately computed locally. Naively computing it locally would effectively result in a stratified Cox model.

To overcome this challenge, we employ a technique known as *survival stacking*. The core idea is that the coefficients in the Cox model approximate those obtained from logistic regression when the dataset is transformed by stacking (4). Since logistic regression has a separable loss function, it can be trained in a federated manner. First, each party locally survival stacks its dataset. The parties then collaboratively perform federated logistic regression using the transformed data. The outcome is an approximation of the Cox model. Its quality can then be assessed using (federated) metrics.

In the following sections, we describe the survival stacking technique in more detail, demonstrate its implementation, and show the process for federated Cox modeling using this approach.

#### 2.2.1 Survival stacking

Survival stacking is a method that transforms a right-censored survival dataset into a classification dataset. This procedure is detailed in (4); here, we provide a brief overview.

Given a survival dataset $D=\left(Z_{i},t_{i},\delta_{i}\right)$, the goal is to construct a classification dataset $D^{\prime }$. The resulting dataset consists of a matrix of independent variables X and a target vector y containing boolean outcomes. The matrix X includes all covariates from D, along with additional columns representing risk set indicators corresponding to each event time point $t_{i}$ where $\delta_{i}=1$. $D^{\prime }$ is built iteratively, by “stacking” the risk sets at the different failure times. More specifically, for each event time point $t_{i}$ where $\delta_{i}=1$, the procedure is as follows:

1. Identify the risk set $R(t_{i})$, which includes all subjects still under observation at time $t_{i}$.
2. For each subject in $R(t_{i})$, add a row to $D^{\prime }$ that replicates their covariates from D.
3. Set the corresponding risk set indicator to 1.
4. Update the target vector y: assign 1 to the subject who experienced the event at $t_{i}$ and 0 to all others.

As an example consider the dataset:

$$D=\begin{array}{ccc} \text{Covariates} & \text{Times} & \text{Event}\\ \left(\begin{array}{c} x_{0}\\ x_{1}\\ x_{2} \end{array}\right), & \left(\begin{array}{c} t_{0}=0\\ t_{1}=1\\ t_{2}=2 \end{array}\right), & \left(\begin{array}{c} \delta_{0}=1\\ \delta_{1}=0\\ \delta_{2}=1 \end{array}\right) \end{array}$$

Since events occur at $t_{0}$ and $t_{2}$ (where $\delta_{0}=1$ and $\delta_{2}=1$), we construct $D^{\prime }$ with three columns: one for the covariate and two for the risk set indicators. At $t_{0}$, the risk set includes all subjects. Each subject is added to $D^{\prime }$ with the first risk set indicator set to 1. The target value is 1 for the event at $t_{0}$ (subject 0) and 0 for others:

$$D^{\prime }=\begin{array}{cc} \text{Covariates} & \text{Target}\\ \left(\begin{array}{ccc} x_{0} & 1 & 0\\ x_{1} & 1 & 0\\ x_{2} & 1 & 0 \end{array}\right), & \left(\begin{array}{c} 1\\ 0\\ 0 \end{array}\right) \end{array}$$

At $t_{2}$, only subject 2 remains in the risk set. We add this subject to $D^{\prime }$ with the second risk set indicator set to 1 and the target value set to 1 (since subject 2 experienced the event at $t_{2}$):

$$D^{\prime }=\begin{array}{cc} \text{Covariates} & \text{Target}\\ \left(\begin{array}{ccc} x_{0} & 1 & 0\\ x_{1} & 1 & 0\\ x_{2} & 1 & 0\\ x_{2} & 0 & 1 \end{array}\right), & \left(\begin{array}{c} 1\\ 0\\ 0\\ 1 \end{array}\right) \end{array}$$

For a more comprehensive explanation of survival stacking and its theoretical justification, see (4). The main result is that applying logistic regression to the resulting stacked dataset provides an approximation of the Cox proportional hazards model coefficients.

#### 2.2.2 Federated logistic regression

Federated learning of logistic regression enables multiple parties to collaboratively train a logistic regression model without sharing their local data. This decentralized approach preserves privacy while allowing the computation of a global model across distributed datasets. In the following, we outline the basic procedure for federated logistic regression.

Consider K parties, each holding a local dataset $D_{k}=\left(X_{k},y_{k}\right)$, where $X_{k}$ represents the feature matrix and $y_{k}$ is the binary target variable. The goal is to jointly estimate the logistic regression parameters β by minimizing the following loss function:

$$L(\beta )=\sum_{k=1}^{K}\sum_{i=1}^{n_{k}}\left[y_{ki}\log(y_{ki}^{\prime })-(1-y_{ki})\log(1-y_{ki}^{\prime })\right],$$

where

1. $n_{k}$ is the number of subjects in $D_{k}$
2. $y_{ki}$ is the ith subject in $y_{k}$, i.e., the true label for ith entry in $D_{k}$.
3. $y_{ki}^{\prime }$ is the prediction for the ith subject in $D_{k}$. This is computed using
  $$y_{ki}^{\prime }=\sigma (X_{ki}^{T}\beta )=\frac{1}{1+e^{-X_{ki}^{T}\beta }}$$

As we can see, the loss function can be computed locally by each party. By averaging the gradients of the local losses, we can compute the gradient of the entire data set. The algorithm starts with an initialization step: a central server initializes the model parameters $\beta^{\left(0\right)}$ and shares them with all participating parties. Next, each party k computes the gradient of the local objective function with respect to β:

$$\nabla L_{k}(\beta )=\sum_{i=1}^{n_{k}}\left[(y_{ki}^{\prime }-y_{ki})X_{ki}\right]$$

The server collects the local updates and takes a weighted average based on the relative size of the data sets. This is the global gradient, which is multiplied by the step size η to compute the global update on the model.

$$\beta^{\left(t+1\right)}=\beta^{\left(t\right)}-\eta \sum_{k=1}^{K}\frac{n}{n_{k}}\nabla L_{k}(\beta^{\left(t\right)}),$$

The updated model is distributed to the different parties. These steps are repeated until convergence has been reached. There are many different ways for the aggregator to determine the step size. In this research, we have tested multiple optimizers, of which the results are given in Section 3.

#### 2.2.3 Federated Cox regression

We can combine survival stacking with federated learning to collaboratively fit a Cox proportional hazards model without sharing raw data. Each participating party independently applies survival stacking to its dataset. Subsequently, the parties engage in a federated logistic regression procedure to fit a logistic regression model on their stacked datasets. The resulting logistic regression coefficients are approximations of the Cox model coefficients. To perform survival stacking, each party must know the time points of the failures. In some cases, these might be considered sensitive data. A solution to this problem is to collaboratively compute the highest time point [by sharing the maximum time point, or using multi -party computation (5)]. Next, we split the time frame into a predetermined number of time points. For each time point, we take the risk set at that point and set the values to 1 for subjects who experience a failure close to that time frame (6). This also reduces the size of the stacked dataset, which can result in more accurate models. In Section 3, we also demonstrate that this method effectively estimates the Cox model coefficients in a federated setting. The code includes the computation of several statistics, such as the Wald statistic and p-values.

### 2.3 Secure Schoenfeld residuals

One of the key assumptions of the Cox model is the so-called *proportional hazards assumption*. The hazard function can be thought of as the risk of an individual having an event at a given time. The proportional hazards assumption now states that this hazard can be split up in two parts: the baseline and a linear combination of the individual’s covariates, and that this baseline is the same for all individuals. Furthermore, it is assumed that both the covariates and the model parameters are time-invariant, i.e., they remain the same over the entire course of the study. Recall from Section 2.1 that the hazard for individual i is given by

$$\lambda (t|Z_{i})=\lambda_{0}(t)\cdot e^{\beta Z_{i}},$$

where $\lambda_{0}(t)$ is the common baseline hazard, $Z_{i}$ are the covariates of the individual and β are the model’s parameters.

Now, this is a very strong assumption that needs to be validated. For this we use Schoenfeld residuals, a concept published by David Schoenfeld in 1982 (7). Simply put, the idea is to compare the actual covariates in the dataset with the covariates predicted by the model. Now, the model does not primarily seek to predict the covariate values, but at the time just before each failure, we can compute the risk-weighted average of the covariate over the relevant risk set and compare that to the actual covariate. We then plot these residuals and inspect them; if they are distributed as random noise, we conclude that the proportional hazards assumption holds, but if there is a clear time dependence in the residuals, we are more likely to reject the assumption.

We have implemented the calculation of Schoenfeld residuals in a secure multi-party computation (MPC) setting, using the MPyC library (8). This calculation is to be performed by the cooperating parties after the federated Cox regression. In order to speed up the calculation as much as possible, the parties can preprocess the data and perform precomputations in order to simplify the actual MPC calculation as much as possible.

#### 2.3.1 Approach

We describe our novel approach to securely compute the Schoenfeld residuals in a federated setting. We assume that every party has access to the trained model coefficients β, as well as their own data. The protocol consists of four steps: preprocessing, sharing of failure times, precomputation, and the MPC calculation.

1. Preprocessing: each dataset contains one event per row. An event can be either a failure or a censoring. Every row also contains the time of the event and the individual’s covariates. In this step, each party perturbs all of their event times by a small random value. This is only done to ensure that there are no duplicate times in the complete dataset. Then the party’s data is sorted by the new perturbed event times.
2. Sharing of failure times: here the parties communicate all of the failure times (including perturbation) in their dataset. Note that these times are generally considered sensitive, as they might leak some information about individuals in case of extremely small datasets. However, the Schoenfeld residuals themselves must be plotted against these failure times, so they are required to be public for the protocol to be useful.
3. Precomputation: this is where most of the computational work is done. Each party (separately) computes the following values for each individual i:
  $$\begin{array}{rl} H_{i} & =\exp\left(\sum_{\;j=1}^{m}C_{ij}\cdot \beta_{\;j}\right),\\ W_{ij} & =C_{ij}\cdot H_{i} \end{array}$$
  where $C_{ij}$ is the jth covariate of individual i, and $\beta =(\beta_{1},\ldots ,\beta_{m})$ are the coefficients of the model for each covariate. For individual i, we call $H_{i}$ the hazard, and $W_{ij}$ the weight for the jth covariate. Next, let $f=(f_{1},\ldots ,f_{K})$ be the vector of sorted failure times shared in the previous step and furthermore $F_{p}$ the set of failures that belong to this party p, then let for each individual i
  $$\begin{array}{rl} HV_{i} & =\sum_{\left\{k\;:\;\;t_{k}\geq f_{i}\right\}}H_{j},\\ WV_{ij} & =\sum_{\left\{k\;:\;\;t_{k}\geq f_{i}\right\}}W_{ij},\\ CV_{ij} & =\left\{ \begin{array}{cc} C_{ij}\; & \text{ if }f_{i}\in F_{p}\;\\ 10pt0\; & 10pt\text{ if }f_{i}\notin F_{p}\;\\ \; \end{array}\right. \end{array}$$
  where $t=(t_{0},\ldots ,t_{n})$ are the times of the events. These values are not very useful on their own, but rather facilitate a simpler calculation in the last step.
4. MPC calculation: following the precomputation, this step is straightforward. However, it happens in the encrypted domain, meaning that none of the inputs is actually visible to other parties. We first present the calculation without encryption:
  $$\begin{array}{rl} TH_{i} & =\sum_{\;p\in P}HV_{i}^{\left(p\right)},\\ TW_{ij} & =\sum_{\;p\in P}WV_{i}^{\left(p\right)},\\ TC_{ij} & =\sum_{\;p\in P}CV_{i}^{\left(p\right)},\\ EC_{ij} & =\frac{TW_{ij}}{TH_{i}},\\ SR_{ij} & =TC_{ij}-EC_{ij} \end{array}$$
  where $SR_{ij}$ is the desired Schoenfeld for individual i and covariate j.

Now, the secure approach involves a technique called secret sharing, in which all parties own a “piece” of each secret value. This means that none of them individually knows anything about the secret (except possibly the party that secret-shared it), but all of them together can reconstruct the secret. The technique is based on polynomial interpolation and was introduced by Adi Shamir in 1979 (9). The additions we do in this protocol are very straightforward under secret sharing, so the main bottleneck is the set of divisions that have to be performed. This is not very complicated, although it does require many communication rounds between the parties.

With this approach, the parties can securely validate the federated Cox model. This is very useful, as this validation is almost always done in practice, but doing it in the clear would nullify the privacy enhancement of the federated training. Hence, being able to do both parts securely makes the whole model more valuable.

#### 2.3.2 Evaluation

**Figure 1:**
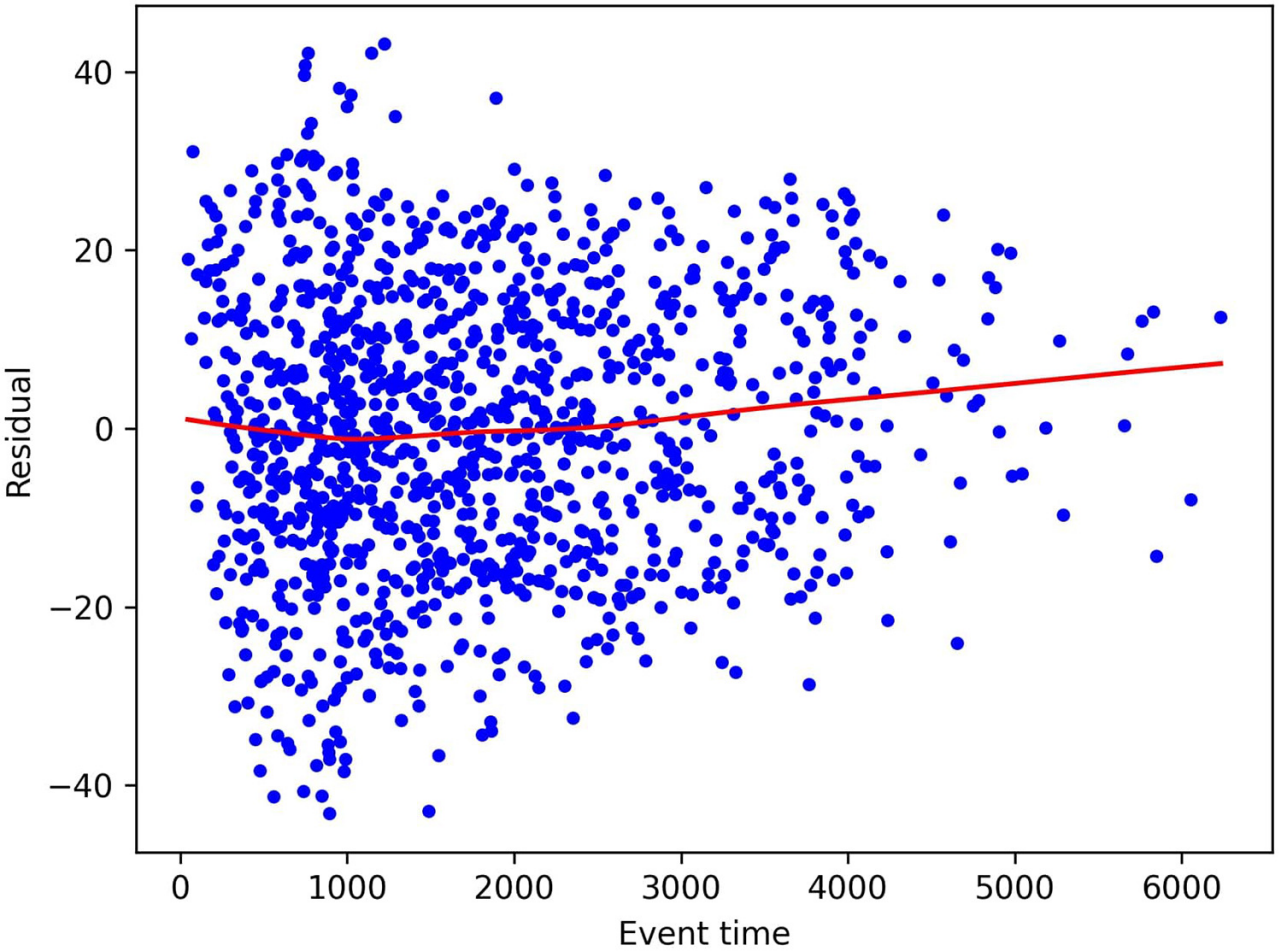
Schoenfeld residuals for the age covariate in the Rotterdam Tumor Bank dataset. The dots represent individual residuals, and the line is a smoothed LOWESS fit.

As mentioned, the standard approach in evaluation Schoenfeld residuals is to plot them and inspect them visually. As an example, we plot the residuals for the age covariate of the Rotterdam Tumor Bank dataset in Figure 1, as computed by the approach described above. We see that the residuals do not show a clear time dependence in this case, also indicated by the fact that the fitted line is close to the constant line through zero. This indicates that indeed the residuals are independently and identically distributed around zero and so the Proportional Hazards Assumption holds. However, we note that this is often a quite subjective measurement and is difficult to quantify exactly.

## 3 Results

### 3.1 Open-source implementation

We provide an open source Python implementation of the described algorithms; see https://github.com/TNO-FL/protocols.cox_regression.

The implementation includes modules for survival stacking, Schoenfeld residuals, and some generic utilities. The main functionality - the training of the distributed Cox model - happens in a client-server model. In simplified terms, the parties (each having a dataset) are separate clients, and the server only facilitates the protocol. The clients all perform an optimization step on their data, send it to the server, who then aggregates these steps to update the global model accordingly, and sends the updated model back to each of the clients. For example, each hospital would run the client and configure the URL of the server, which is usually run by an independent third party. The implementation details and recommended usage are also described in the repository.

### 3.2 Experiments

In this section, we present various experiments conducted to evaluate the performance of the proposed algorithm. By comparing our approach to established reference implementations, we can better quantify any inaccuracies introduced by survival stacking relative to directly applying a Cox model. In Section 3.2.1 we compare different optimizers against a reference implementation, then in Section 3.2.2 we analyze the impact of survival stacking. Next, Section 3.2.3 motivates the applicability by demonstrating both separate and collaborative models, and finally Section 3.2.4 presents the accuracy of both survival stacking and federated learning in the context of the Cox model.

#### 3.2.1 Comparison of optimizers

We begin by comparing the effectiveness of different optimization methods for fitting a logistic regression model on stacked data. Survival stacking significantly increases the size of the dataset, resulting in a high proportion of zero values in the risk set indicator columns. Furthermore, the target vector contains very few positive labels, leading to a sparse dataset with a highly imbalanced class distribution. As a consequence, a model that sets all coefficients to zero already achieves a high accuracy score. To assess the performance of our approach, we therefore compare it against the coxph implementation in the lifelines software package (10). We compare with a reference implementation, instead of using scores like a C-index or calibration plots, as evaluating the accuracy of a Cox model purely through numerical metrics is challenging and often requires contextual and domain knowledge. By comparing our approach to established reference implementations, we can better quantify any inaccuracies introduced by survival stacking relative to directly applying a Cox model.

The results of our evaluation on two datasets are presented in Tables 1 and 2. Each experiment was repeated 10 times until convergence and the results were subsequently averaged.

**Table 1:** The outcomes for several solvers on the Rotterdam Tumor Bank dataset.

Solver	age	grade	node	pgr	er	meno	hormon
Lifelines (10)	0.0184	0.3772	0.0881	−0.0004	−0.0001	−0.0369	−0.0388
Gradient Descent	−0.0388	−0.7026	0.0883	−0.0013	−0.0006	0.4068	−0.0023
Momentum	−0.0303	−0.7117	0.1086	−0.2274	−0.2481	0.2179	0.1404
Adam	0.0185	0.3807	0.0936	−0.0004	−0.0001	−0.0298	−0.0699
Newton Cholesky	0.0184	0.3780	0.0928	−0.0004	0.0000	−0.0313	−0.0632

Lifelines is the reference implementation. The other rows are obtained by fitting logistic regression on a central stacked dataset with 50 time bins. For gradient descent and momentum ran 100.000 iterations with lr=0.001 and v=0.9. For Adam, we used 50.000 iterations and $\left(lr,\beta_{1},\beta_{2})=(0.001,0.9,0.999\right)$. Newton–Raphson converged in less than 25 rounds.

**Table 2:** The outcomes for several solvers on the Colon dataset.

Solver	sex	age	obstruct	perfor	adhere	nodes	surg
Lifelines (10)	−0.1512	−0.0041	0.2209	0.2107	0.2603	0.0872	0.2717
Gradient Descent	−0.0392	−0.6952	0.0880	−0.0013	−0.0003	0.4120	0.0228
Momentum	−0.4718	−0.0650	−0.2323	0.0090	0.2597	0.0583	0.0774
Adam	−0.1465	−0.0043	0.2214	0.2162	0.2713	0.0945	0.2762
Newton Cholesky	−0.1425	−0.0042	0.2198	0.2108	0.2708	0.0961	0.2752

Lifelines is the reference implementation. The other rows are obtained by fitting logistic regression on a central stacked dataset with 50 time bins. For gradient descent and momentum ran 100.000 iterations with lr=0.001 and v=0.9. For Adam, we used 50.000 iterations and $\left(lr,\beta_{1},\beta_{2})=(0.001,0.9,0.999\right)$. Newton–Raphson converged in less than 25 rounds.

The findings indicate that both the Adam optimizer and the Newton–Raphson method converge to values close to the reference Cox parameters. Adam requires a greater number of communication rounds, whereas Newton–Raphson involves transmitting the Hessian matrix, which can be large. The choice between these methods thus represents a trade-off between the frequency and the size of communication. This trade-off largely depends on the size of the Hessian matrix, which is primarily influenced by the number of time bins used in the analysis.

#### 3.2.2 Accuracy of survival stacking

We also evaluate the accuracy of our survival stacking approach, first in a centralized setting and then in a federated one. Rather than evaluating our implementation using independent measures, we benchmark our results against reference implementations in lifelines and R.

A key factor influencing the accuracy of survival stacking is the number of time bins used. In the original stacking approach, a new stack was added for each failure event. However, this strategy can lead to excessively large models, which is impractical, particularly in the context of federated learning. To address this issue, we employ time binning, as described in Section 2.2.3. We therefore evaluate the performance of survival stacking across different numbers of time bins. The results for various datasets are presented in Tables 3 and 4.

**Table 3:** The results for the Rotterdam Tumor Bank dataset in a central setting for different number of bins.

# bins	age	grade	node	pgr	er	meno	hormon	Distance
Lifelines (10)	0.0184	0.3772	0.0881	−0.0004	−0.0001	−0.0369	−0.0388	
1	0.0186	0.4127	0.1887	−0.0004	0.0002	0.0581	−0.6878	0.6646
10	0.0184	0.3926	0.1113	−0.0004	0.0000	0.0009	−0.1718	0.1411
25	0.0186	0.3832	0.0974	−0.0004	0.0000	−0.0276	−0.0939	0.0569
50	0.0184	0.3780	0.0928	−0.0004	0.0000	−0.0313	−0.0632	0.0255
75	0.0185	0.3761	0.0914	−0.0004	−0.0001	−0.0355	−0.0603	0.0218
100	0.0182	0.3740	0.0903	−0.0004	−0.0001	−0.0323	−0.0555	0.0178
300	0.0181	0.3704	0.0883	−0.0004	−0.0001	−0.0342	−0.0509	0.0141
400	0.0180	0.3693	0.0879	−0.0004	−0.0001	−0.0331	−0.0507	0.0148
500	0.0179	0.3684	0.0876	−0.0004	−0.0001	−0.0328	−0.0512	0.0158

The distance is the Euclidean distance from the reference implementation.

**Table 4:** The results for the colon dataset in a central setting for different number of bins.

# bins	age	grade	node	pgr	er	meno	hormon	Distance
Lifelines (10)	−0.1512	−0.0041	0.2209	0.2107	0.2603	0.0872	0.2717	0.0000
1	−0.1027	−0.0055	0.2131	0.2545	0.4824	0.1818	0.4012	0.2817
10	−0.1347	−0.0044	0.2212	0.2452	0.2883	0.1203	0.3074	0.0680
25	−0.1350	−0.0044	0.2235	0.1996	0.2783	0.1026	0.2803	0.0320
50	−0.1431	−0.0042	0.2224	0.2043	0.2651	0.0966	0.2768	0.0157
75	−0.1445	−0.0041	0.2205	0.2051	0.2670	0.0946	0.2754	0.0138
100	−0.1452	−0.0040	0.2215	0.2079	0.2658	0.0937	0.2759	0.0116
200	−0.1465	−0.0040	0.2185	0.2171	0.2679	0.0936	0.2792	0.0150
300	−0.1470	−0.0040	0.2186	0.2187	0.2721	0.0942	0.2813	0.0192
400	−0.1473	−0.0041	0.2166	0.2223	0.2733	0.0947	0.2845	0.0236
500	−0.1476	−0.0041	0.2168	0.2248	0.2752	0.0953	0.2860	0.0269
No binning	−0.1473	−0.0041	0.2179	0.1932	0.2542	0.0860	0.2646	0.0205

The distance is the Euclidean distance from the reference implementation.

We see that more time bins lead to better results, up to a certain value. Introducing more time bins beyond that point leads to a more sparse dataset, without adding much more information. For both the Rotterdam and Colon dataset, the optimal number of time bins was 100. The optimal number of bins also depends on the size of the dataset. Interestingly, time-binning can result in models closer to the reference Cox model than the original stacking method.

#### 3.2.3 Benefit of federated learning

**Figure 2:**
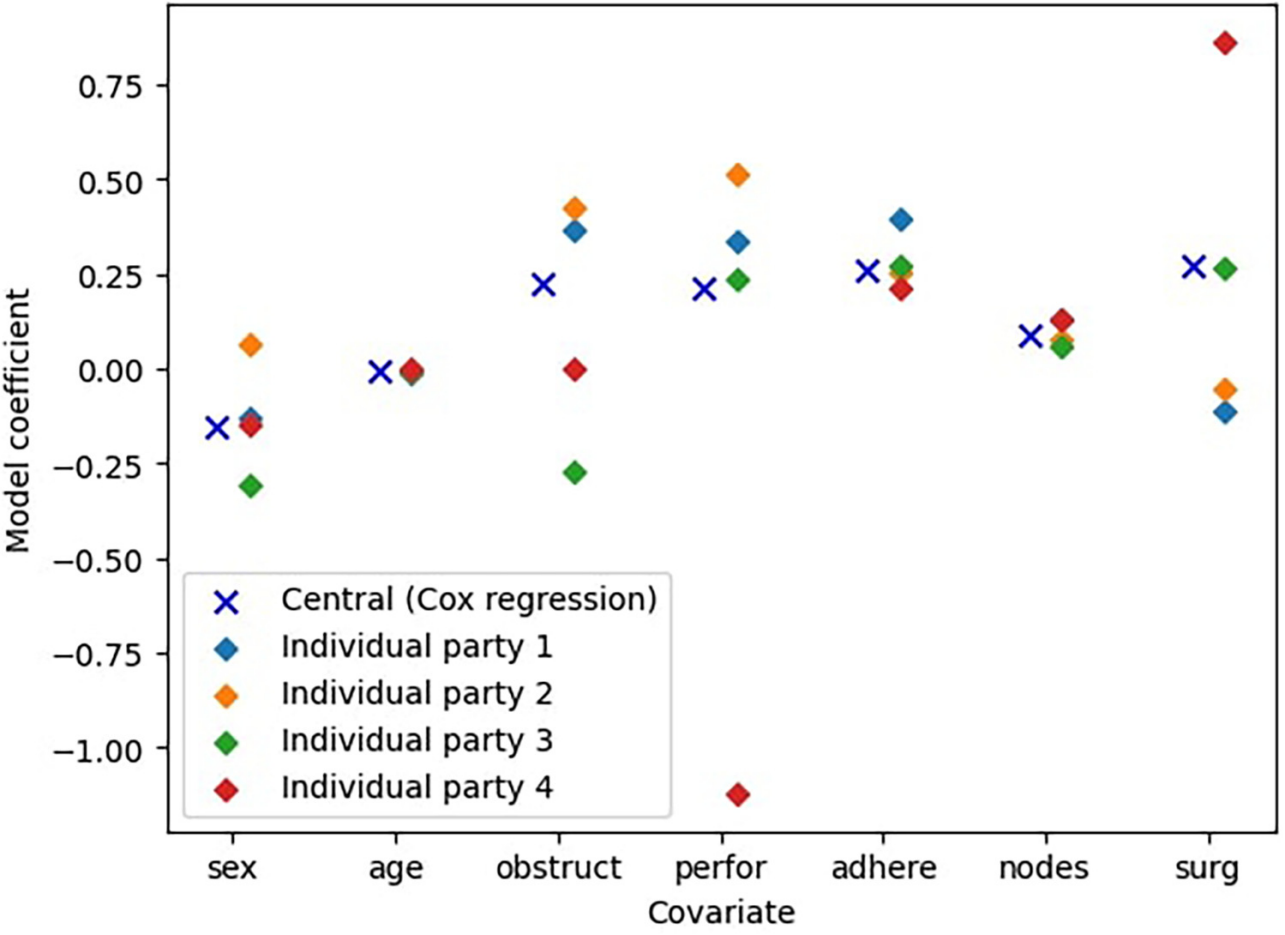
Comparison between the central Cox model and the models trained only on an individual parties’ data (n=4 parties).

In this experiment, we evaluate the advantages of federated learning over training a Cox model locally. We use the colon dataset (11) and split the dataset uniformly at random, so that every party has nearly the same number of data points. To ensure robustness, we repeat the experiment 10 times with different splits. The results are in Table 5. Examples are also shown in Figure 2, which presents partial results visually.

**Table 5:** Comparison in accuracy loss for federated and individual models.

Exp.	n=2		n=3		n=4		n=5	
	Fed.	Ind.	Fed.	Ind.	Fed.	Ind.	Fed.	Ind.
1	0.086692	0.508615	0.186127	0.763789	0.301681	1.298265	0.367590	1.793961
2	0.085923	0.253846	0.090872	0.750492	0.233104	1.333657	0.221824	1.318142
3	0.105217	0.925193	0.150346	1.014060	0.118710	1.258620	0.197675	1.339609
4	0.094011	0.861348	0.183629	1.191052	0.396855	1.530512	0.318490	1.325883
5	0.074628	0.912278	0.213943	1.117565	0.229633	1.535407	0.269670	5.035956
6	0.085935	0.607380	0.192031	0.696788	0.228756	1.167630	0.243405	1.397925
7	0.094830	0.513158	0.242559	1.691819	0.174044	1.454943	0.450566	1.888442
8	0.082531	0.698606	0.206044	1.099592	0.162499	1.337512	0.320388	1.318446
9	0.085296	0.373202	0.214295	1.094962	0.306755	1.179061	0.551078	4.566361
10	0.097963	0.783727	0.142877	0.875390	0.317852	1.532268	0.353232	4.702105
avg.	0.089302	0.643735	0.182272	1.029550	0.246988	1.362787	0.329391	2.468683

Each row displays results of a different experiment with randomly split data. For 2≤n≤5 parties, both models are compared to the ideal case of the central Cox model, and the accuracy loss is measured as the Manhattan distance of the model’s parameters to those of the ideal model. A lower value therefore means a better approximation of the ideal model. The last row contains the average of the experiments. The colon dataset was again used.

In Table 5, we assess the benefits of federated learning in terms of model accuracy, by performing a comprehensive comparison. This table presents the accuracy loss of the federated models alongside that of individually trained models. To ensure a fair comparison, each pair of federated and individual models (corresponding to the same row and number of parties) is trained on identical data partitions.

We measure the accuracy of a model by comparing it to the ideal central Cox model (trained on all of the combined data) and take the accuracy loss to be the Manhattan distance between the coefficients of both models. That is, let $\beta^{*}=(\beta_{1}^{*},\ldots ,\beta_{m}^{*})$ be the optimal coefficients of the central Cox model, and consider some other model with coefficients $\beta =(\beta_{1},\ldots ,\beta_{m})$, then we define that model’s accuracy loss as

$$\sum_{i=1}^{m}|\beta_{i}-\beta_{i}^{*}|.$$

A lower value corresponds to a better approximation of the optimal model. We opt for this approach since we wish to assess how well we can fit a Cox model in a federated setting. Therefore, we compare with a reference implementation, instead of using scores like a C-index or calibration plots. Additionally, evaluating the accuracy of a Cox model purely through numerical metrics is challenging and often requires contextual and domain knowledge.

Our findings show that federated models consistently yield results significantly closer to those of the central Cox model than their individually trained counterparts. Although some variance is observed, primarily due to randomness in data partitioning, the accuracy loss in the individual models is approximately 5 to 8 times greater than that of the corresponding federated models. Moreover, for both federated and individual models, accuracy loss increases with the number of parties. This is likely due to the fixed size of the total dataset: as n increases, the data available to each individual party decreases, making it more challenging to fit accurate models. However, in a real-world scenario, adding more parties would typically contribute additional data rather than merely redistributing a fixed dataset. As a result, we expect that increasing the number of parties in practice would enhance model quality by incorporating more diverse information.

Furthermore, Figure 2 shows a particular example to illustrate the difference between the models. We see that in this case the individual parties’ best models are quite inaccurate and in most cases not comparable to the ideal centralized model. For instance, the model trained by Party 2 overestimates the coefficients for the covariates sex, obstruct, and perfor, while underestimating the coefficient for surg. Consequently, this misestimate leads to over- and underestimation of the effects of these covariates on survival probabilities, resulting in less reliable predictions. This highlights that models trained on a limited subset of the data generally perform significantly worse than those trained on the complete dataset. This demonstrates the clear benefits of collaborative approaches such as federated learning, which enable improved model performance without requiring data centralization.

#### 3.2.4 Accuracy of federated Cox regression

We now evaluate the accuracy of the federated Cox regression model. Survival stacking and data federation can both lead to inaccuracies in the outcome model. Here we assess this loss in accuracy, by testing it on the colon dataset with 25 bins.

**Figure 3:**
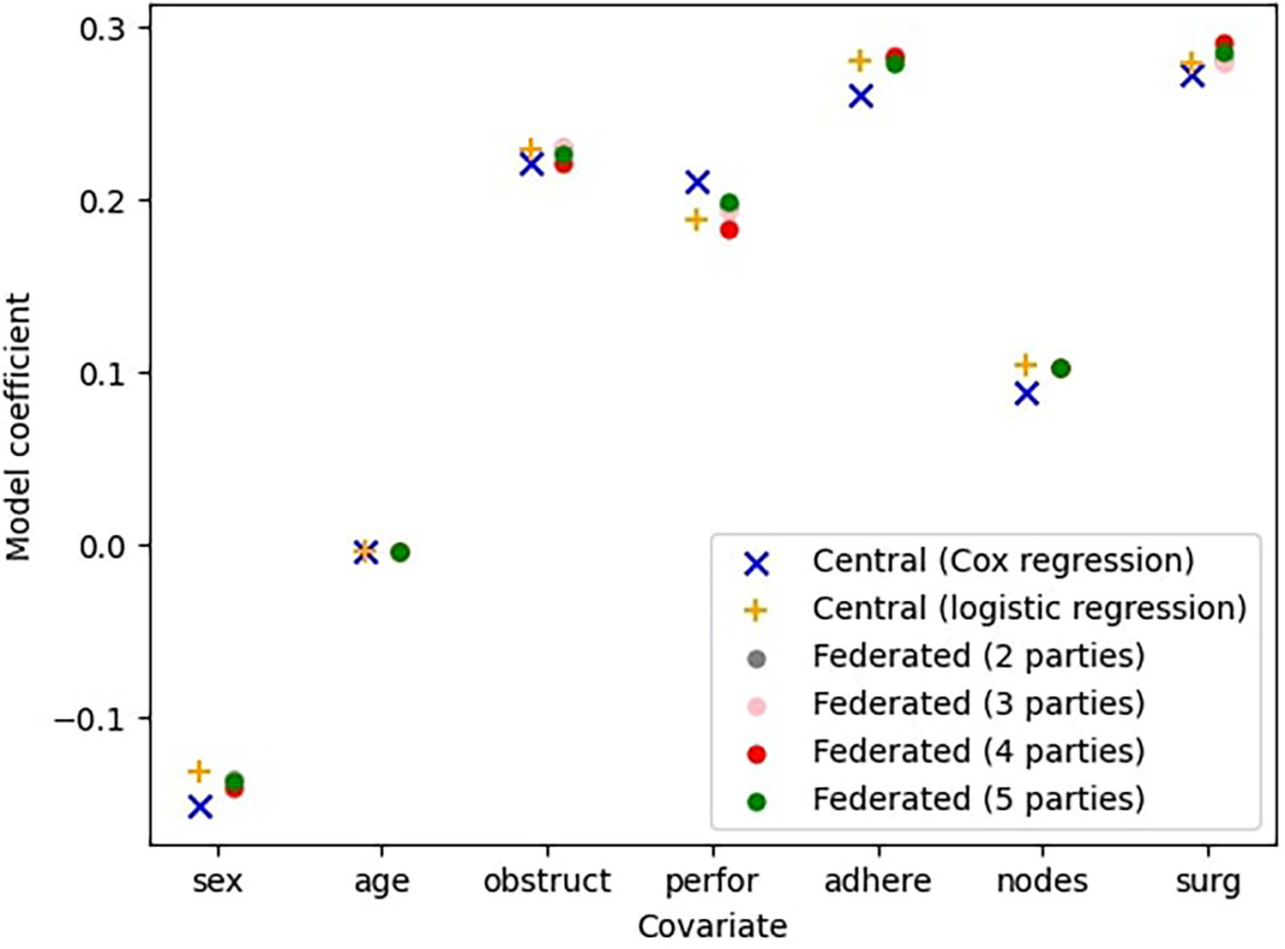
Comparison between the central (ideal) model, the model with survival stacking, and models trained by federated learning with 2≤n≤5 parties.

Figure 3 presents the results of the central Cox model and the central logistic regression model, both of which assume a fully centralized setting where all data is aggregated in a single location. Alongside these, it shows the outcomes of the federated Cox regression for varying numbers of participating parties. Note that the central Cox model is the same as in Figure 2. However, while Figure 2 examines individual Cox models trained by a single party on a fraction of the complete dataset (with a fixed number of parties, n=4), here we evaluate federated models trained across all n parties, where 2≤n≤5.

This figure indicates that the federated models closely approximate the centrally trained Cox model. Furthermore, the sources of error introduced by survival stacking and federated learning do not necessarily compound. For example, for the covariate sex, the central logistic regression model overestimates the coefficient relative to the central Cox regression model, whereas the federated regression model underestimates it relative to the central logistic regression model. This interaction results in a slight improvement in the overall estimation. The p-values give an indication of the error.

Our results demonstrate that federated models produce significantly more accurate predictions than models trained independently by individual parties. These findings underscore the benefits of collaborative model training, particularly in scenarios where direct data sharing is not feasible. Federated learning thus presents a viable approach for improving Cox model accuracy while preserving data privacy.

## 4 Discussion

### 4.1 Related work

The Cox model has been extensively studied in the scientific community [see, e.g., (12) for an overview], and several approaches have been proposed for fitting a Cox model in a federated setting using privacy-preserving methods. Some studies employ statistical learning techniques (6, 13, 14), while others utilize secure multi-party computation (MPC) (15) or related cryptographic techniques (16). Furthermore, the combination of survival stacking with federated learning has been explored in previous work, often in conjunction with custom classifiers rather than logistic regression (17–19).

However, many of these existing solutions involve sharing time-to-event information between participating institutions. In our approach, we treat time as a privacy-sensitive variable during the learning phase and ensure that it is not shared. Some alternative methods also avoid sharing time, but instead report only model performance metrics such as the concordance index (c-index). Our findings indicate that multiple distinct models can yield the same c-index, and furthermore, the c-index is not a reliable performance metric in all scenarios (20). To address this limitation, we compare models trained using our distributed approach against known models from the centralized setting.

Furthermore, we incorporate statistical significance testing through p-value computation and implement a commonly used diagnostic tool in medical research: Schoenfeld residuals. To the best of our knowledge, this is the first study to compute Schoenfeld residuals on distributed data.

### 4.2 Performance

While our proposed approach yields very significant privacy and security benefits, this comes at the cost of computational complexity. It is clear that these privacy-preserving methods (in this case federated learning and MPC) introduce some additional overhead, both in computational effort and communication time, compared to a centralized approach. That being said, federated learning does not introduce a large amount of overhead, as the protocol only requires parties to communicate relatively small updates to the model, and the dominating computation is still calculating these updates (which is required regardless of federated learning). MPC introduces comparatively more overhead, but arguably this is still manageable. Specifically because the computation in question (that of Schoenfeld residuals) is not extremely complex. Furthermore, we see in general that training a model such as the Cox Proportional Hazards Model is not a time-constrained task; often there is quite some time available and it is not vital for the training process to complete in seconds. In this way, the additional overhead is not as destructive as it is in some other applications. Finally, we argue that this drawback does not weigh up against the benefit of added privacy, specifically because we are considering sensitive patient data. In fact, using a central model is theoretically better than using a federated model, but the latter is practically much better than not collaborating with data at all.

### 4.3 Further research

Our approach focuses on horizontally partitioned data, where each individual is associated with a single party that has access to all covariates for that individual. In contrast, vertically partitioned data refers to a setting in which all parties have records for the same individuals but possess only a subset of the covariates. Horizontal partitioning is more straightforward to implement in a federated learning framework, as each party can independently evaluate individuals and observe their respective events. In a vertically partitioned setting, however, no single party has access to both the outcome events and the full set of covariates, making model training significantly more complex. Additionally, survival stacking is also requires communication between the parties. The complexity and development of vertically federated Cox regression using survival stacking remains an open research problem.

In this study, we provide model assessment by incorporating p-values and Schoenfeld residuals in the code. However, in the evaluation of medical models, additional validation techniques, such as calibration plots, are commonly used in practice. Implementing these methods in a privacy-preserving manner could further improve the applicability and robustness of our approach.

Additionally, the number of time bins is a critical parameter in survival stacking, directly influencing model accuracy. Selecting an appropriate number of bins is essential for reliable estimation. Developing methods to determine the optimal number of bins prior to model training would be a valuable extension to this work, improving both efficiency and predictive performance.

## Data Availability

Publicly available datasets were analyzed in this study. This data can be found here: https://search.r-project.org/CRAN/refmans/condSURV/html/colonCS.html.
